# Acute Arthritis in Children: How to Discern between Septic and Non-Septic Arthritis?

**DOI:** 10.3390/children8100912

**Published:** 2021-10-13

**Authors:** Lisa Gamalero, Giovanna Ferrara, Teresa Giani, Rolando Cimaz

**Affiliations:** 1 Department of Medicine, University of Udine, 33100 Udine, Italy; lisa.gamalero@gmail.com; 2 AUSL Toscana Centro, 50122 Florence, Italy; giovanna.ferrara@gmail.com; 3 Department of Clinical Sciences and Community Health, University of Milan, 20122 Milan, Italy; rolando.cimaz@unimi.it

**Keywords:** arthritis, acute, septic, children, differential diagnosis

## Abstract

The term septic arthritis refers to an infection of the synovial space. This is an infrequent condition in healthy children, but it should be considered a medical emergency potentially leading to irreversible articular damage. Therefore, prompt diagnosis and antimicrobial treatment play a crucial role in improving the prognosis. Although septic arthritis is the most common cause of acute arthritis, many other diseases may mimic a similar clinical picture, constituting a diagnostic challenge for the clinician who first approaches the patient. Herein we analyze the main features of septic arthritis, offering an overview of the main conditions involved in the differential diagnosis and suggesting a diagnostic workup plan.

## 1. Introduction

Septic arthritis is a medical emergency potentially leading to a rapid morbidity with irreversible articular damages; it represents one of the most concerning causes of arthritis.

The term arthritis refers to an inflammation of the joint synovium regardless of its etiology. When inflammation lasts less than 6 weeks, arthritis is defined as acute. Although the most common cause of acute arthritis, especially of monoarthritis, is an infection of the joint space, known as septic arthritis, a broad differential diagnosis might represent a diagnostic challenge for the clinician who first approaches the patient. In a recent report by Thomas et. al., 196 patients aged between 3 months and 6 years with acute monoarticular inflammation were evaluated: 56.1% had a septic arthritis, 10.2% had onset of juvenile idiopathic arthritis, and for 33.7% no definitive diagnosis was possible [1].

Septic arthritis mostly affects children younger than 4 years of age, with an overall incidence estimated to be 4–10 per 100,000 children in industrialized countries [2]. Hematogenous dissemination of bacteria, seldom fungi, mycobacteria, and viruses, or less commonly a contiguous spread from surrounding soft tissues or a direct inoculation into the joint of infectious agents (e.g., penetrating accidental trauma or iatrogenic procedures such as arthrocentesis or arthroscopy), can lead to joint infection [3]. Septic arthritis is usually monoarticular, and hip and knee are the joints most frequently involved. A polyarticular distribution should always be suspected in neonates and immunocompromised patients. *Staphylococcus aureus* is considered the most frequent pathogen responsible for osteomyelitis and septic arthritis in any age group, mainly methicillin-sensitive strains (MSSA), as it is accountable for up to 70–90% of confirmed cases. Nevertheless, an update on the microbiologic causes led to the current knowledge that different pathogens can be found depending on the children’s age, comorbidities, immune and vaccination statuses, and socioeconomic conditions [4]. In very young children and toddlers, Streptococcus agalactiae and other Gram-negative organisms (e.g., *Neisseria meningitidis*, *Neisseria gonorrhoeae*, *Escherichia coli*) and occasionally *Staphylococcus aureus* are recognized as potential pathogens. In children between 2 and 5 years of age, *Streptococcus pyogenes* and *Streptococcus pneumoniae* should also be considered [4,5]. *Kingella kingae*, a Gram-negative bacterium, is an emerging pathogen representing in some series over 80% of the causes in patients between 28 days and 4 years of age. [1,6]. Samara et al. compared bacteriologic data of patients with osteoarticular infections over 20 years, dividing them in two groups before and after the extensive use of nucleic acid amplification assays in the diagnostic process. In the second group, where the molecular technique was used extensively, *Kingella kingae* was the most frequently isolated pathogen (51% of the confirmed cases) with an increase especially in children aged 6 to 48 months. Of note, the molecular technique overall improved the detection of the bacteria responsible for the infection, with 78.4% of identified cases compared to 59.4% during the first conventional period [7]. *Pseudomonas aeruginosa* and *Streptococcus pyogenes* could be responsible for infection after a trauma or surgery, while *Salmonella* spp. can be found in patients with drepanocytosis. *Mycobacterium tuberculosis*, *Bartonella henselae*, and fungi (i.e., *Histoplasma* spp. and *Cryptococcus* spp.) should be considered mainly in immunocompromised children [4].

Clinical presentation varies depending upon the age of the child, the site of infection, and the causative organism. Typically, the disease onset is acute with fever, severe joint pain, swelling, and limited range of motion, and the skin over the joint may appear red and warm. In some patients, especially if very young, the pain may be referred to other structures, such as the knee in hip arthritis, or abdominal pain in sacroiliac joint involvement that might present itself with symptoms mimicking appendicitis, pelvic neoplasm, or urinary tract infection [8,9]. Neonates and young infants may show mild signs and symptoms that can induce a delay in diagnosis. Some pathogens may induce an indolent infection such as *Kingella kingae* that often is responsible in young children with a pauci-symptomatic articular infection associated with mild increased/normal WBC and CRP values, while ESR and platelet counts better reflect the osteoarticular infectious condition [10]. Irritability, poor feeding, pseudoparalysis, aversion to or apparent discomfort on being handled; postural changes; and unilateral swelling of the extremities, buttocks, or genitalia are frequently indirect signs of an acute arthritis in early childhood [11,12,13]. Unlike neoplasms and inflammatory conditions, in osteoarticular infections usually there is only one joint involved, the child is ill-appearing and gets worse quickly if left untreated, up to septic shock. Moreover, except for the fever, there are no other systemic features (e.g., rash, liver and spleen enlargement, lymphadenopathy, and weight loss) associated.

Blood tests are the very first actions to take when septic arthritis is suspected. Thomas et al. found that CRP levels, hemoglobin, and WBC counts were significantly different in patients with septic arthritis compared to those ones with juvenile idiopathic arthritis and undefined arthritis [1]. Moreover, CRP is an excellent negative predictor factor for septic arthritis and is more used in clinical practice compared to ESR [14,15]. In fact, CRP usually peaks within 36–50 h from the infection onset and falls to normal within a week of successful treatment, so it can be used also for monitoring the course of the disease. ESR instead may remain elevated for up to 30 days [16]. Blood and joint fluid cultures should be required in the suspicion of septic arthritis before starting antibiotic therapy, even if they confirm the diagnosis only in 34–82% of cases [17,18,19]. Aspiration of synovial fluid should be performed as soon as possible also as a component of treatment, since the removal of synovial fluid decompresses the joint giving relief from pain. Synovial fluid analysis usually reveals a white blood cell count (WBC) of more than 50,000 cells/microL, with a predominance of polymorphonuclear leukocytes. In the aforementioned cohort of 196 pediatric patients with monoarthritis, WBC count > 50,000 cells/mm^3^ was found in 72.7% of patients with septic arthritis, more frequently compared to JIA (44.4%) and undefined arthritis (34.6%) [1]. Other studies in the literature confirm the same data, also in adult populations [20,21]. Nevertheless, WBC can be less than 50,000 cells/microL in patients with unusual causes of bacterial arthritis (e.g., *Brucella*) and may exceed 50,000 cells/microL in children with juvenile idiopathic arthritis [22,23,24]. Moreover, a positive Gram stain of the synovial fluid is found in less than half of cases [25]. Specific PCR for bacteria on synovial fluid may be useful in the case of negative cultures, since it is more sensitive and specific than standard culture and Gram stain [26]. Ceroni et al. also investigated the use of oropharyngeal swab (PCR) to predict osteoarticular infection due to *Kingella kingae* in young children with success, so this might be an adjuctive tool in doubtful cases [27]. Therefore, at first instance, an acute arthritis should be considered a septic arthritis until proven otherwise; the combination of patient history, laboratory, clinical, and imaging findings should support the clinicians in differential diagnosis.

Although laboratory tests are mandatory, it has been demonstrated that in children less than 6 years of age with acute monoarthritis, the clinical and biological parameters currently used do not reliably differentiate between septic arthritis and JIA or undifferentiated arthritis, and often only evolution can differentiate them [1]. In fact, in case of increased WBC, ESR, and CRP that could be elevated both in septic and inflammatory arthritis, the response to antibiotic drugs and the evolution within the first 48 h will guide the clinician to confirm the correct diagnosis. On the other hand, if there is no response to antibiotic treatment and a progressive alteration in the complete blood count is detected, acute leukemia has to be suspected and excluded.

Imaging offers additional information in the case of suspected septic arthritis, especially for those joints that are not so easy to evaluate with physical examination. Radiographic abnormalities are usually absent or inconclusive in the acute setting; an increased joint space can be appreciated in cases of joint effusion. Radiography may be helpful in identifying concomitant osteomyelitis and evaluating other conditions in the differential diagnosis (e.g., trauma, neoplasia). Ultrasonography and MRI are both sensitive in detecting and quantifying joint effusion and can reveal synovial thickening. Ultrasonography is more widely and readily available, less costly, does not require sedation in young children, and it is also helpful to guide diagnostic aspiration. On the contrary, MRI (especially fat-suppressed, fluid-sensitive and contrast-enhanced sequences) gives more information on extra-articular tissues and is the key exam for differential diagnosis. CT-scan is seldom used, but it may add information in the case of concomitant osteomyelitis, high suspicion of osteoid osteoma not detected with MRI, and failure to respond to initial management. Although MR is able to offer detailed anatomical data without exposure to ionizing radiations, nuclear medicine such as positron emission tomography can still find a place in the diagnostic work-up of musculoskeletal infections as in the presence of a clinical suspicion, but negative MR or in the impossibility to perform MR. Bone scintigraphy combined with labelled white blood cells scintigraphy may localize and define the extent of the infection, especially in deep joint involvement or concomitant osteomyelitis [28].

Regarding prognosis, septic arthritis is a very severe disease for its potentially devastating complications, including osteomyelitis, meningitis, abscess formation, and septic shock, in addition to the destruction of the articular cartilage and ossification centers. Prompt diagnosis and treatment are associated with an excellent prognosis. Initial management of this disease includes broad-spectrum intravenous antibiotics, joint aspiration, and eventually surgical drainage [29]. Any empirical therapy should include coverage of *S. aureus*. When community-acquired MRSA prevalence is 10–15% or higher, this pathogen should be included in the choice of empiric antibiotic therapy. Local, up-to-date resistance patterns are required to decide the best initial empirical therapy. Beta-lactams, such as first and second generation cephalosporins or other antistaphylococcal penicillins, are the drugs of choice for good experience and tolerance. Clindamycin can be added if community-acquired MRSA is a possible cause [30]. Intravenous antibiotic treatment should be provided for the least period of time and switched to oral therapy as soon as physical evaluation and inflammatory markers have improved, for a whole duration of 2/3 weeks [4]. Consultation with an expert in pediatric infectious diseases and/or pediatric rheumatology may be helpful in difficult cases. Orthopedic evaluation should be required in the case of no antibiotic response, especially for arthritis of the hip joint or shoulder [31,32,33,34]. Given the potential morbidity of delayed treatment, children with probable bacterial arthritis should be managed in the same manner as children in whom infection has been confirmed by isolation of an organism from synovial fluid.

## 2. Differential Diagnosis

Many different conditions can mimic septic arthritis in their presentation, making diagnosis difficult. A thorough history and detailed physical examination are essential. The presence of precipitating factors, such as a history of trauma, could be suggestive for a fracture, hemarthrosis or osteomyelitis, while a recent illness, especially with enteric pathogens (e.g., *Salmonella*, *Shigella*, *Yersinia*, and *Campylobacter*), viruses (e.g., *Parvovirus* B19 and *Varicella*) or an immunization (e.g., rubella immunization), may be associated with reactive arthritis. Pain characterization, including site; number of joints involved; and the severity, frequency, duration, and pattern of pain may discriminate between acute and chronic conditions.

On physical examination, the joints must be evaluated for crepitus, warmth, tenderness, swelling, joint effusion, erythema, contractures, or decreased range of motion. The patient’s preferred position at rest must be observed. The joints should be tested for passive and active range of motion. Examination of the contralateral joints is important for comparison purposes. The patient’s gait should be carefully observed. An acute onset of illness with an inflamed painful joint and restricted range of movement clinically indicates septic arthritis unless proved otherwise.

Signs and symptoms of serious conditions should be investigated. Fever, and skin findings such as erythema, purpura/petechiae could be caused by many rheumatic conditions and tumors.

**Figure 1:**
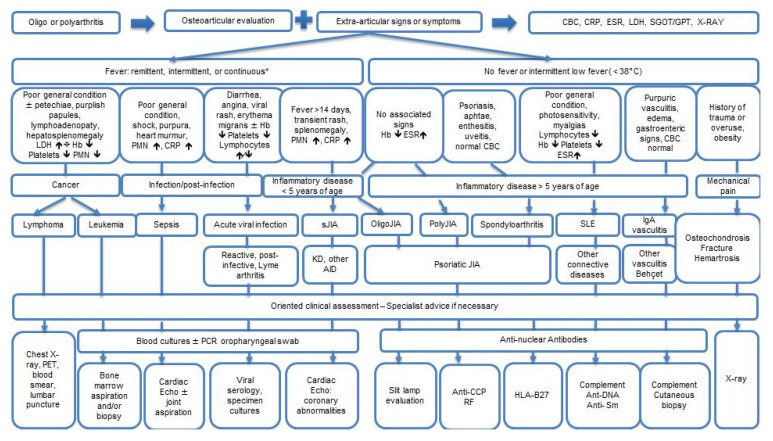
Flow chart showing the differential diagnosis of acute onset arthritis in children. * = except for *Kingella kingae*, see in the text. CBC: complete blood count. CRP: C-reactive protein. ESR: erythrocyte sedimentation rate. LDH: lactate dehydrogenase. SGOT: serum glutamic-oxalacetic transaminase. GPT: serum glutamic pyruvic transaminase. PMN: polymorphonuclear leukocytes. sJIA: systemic juvenile idiopathic arthritis. OligoJIA: oligoarticular juvenile idiopathic arthritis. PolyJIA: polyarticular juvenile idiopathic arthritis. SLE: systemic lupus erythematosus. KD: Kawasaki disease. AID: autoinflammatory disease. PET: positron emission tomography. PCR: polymerase chain reaction. ↑, increased. ↓, decreased. Cardiac echo: echocardiography.

Below we present a brief outline of the main disorders in differential diagnosis with septic arthritis. Figure 1 summarizes the differential diagnostic pathway for acute arthritis in children.

### 2.1. Orthopedic Conditions

Orthopedic conditions may be suspected in some cases, especially if there is a history of recent trauma and/or sports activity. Osteochondrosis is a disease of the growth cartilage endplate or ossification centers, characterized by localized bony necrosis and subsequent regrowth of the bone. Its etiology is probably due to repetitive strain and overuse injury on the growth cartilage, with a genetic background. The most frequent sites involved include the patellar tendon attachment at the tibia, named Osgood-Schlatter disease, the calcaneus (i.e., Sever disease), and the femoral head (i.e., Legg-Calvé-Perthes disease). Other sites include the second metatarsal head (i.e., Freiberg disease), navicular bone (i.e., Köhler bone disease), capitellum (i.e., Panner disease) spine (Scheuermann’s disease), and tibia (Blount disease) [35,36]. The diagnosis is clinical, characterized by chronic and mechanical pain in the typical sites without systemic symptoms and normal laboratory tests; radiographs of the painful site help to confirm it. The treatment usually consists of relative rest and physical rehabilitation and limited use of nonsteroidal anti-inflammatory drugs. Osteochondritis dissecans (OCD) is a disorder of the joint that affects the articular surface and the subchondral bone and potentially leads to detachment of cartilage and bone fragment in the joint space. OCD typically presents with poorly localized, activity-related pain. Crepitus, catching, or locking of the joint may occur during the later stages.

Diagnosis is made with the combination of patient history, clinical presentation of bone pain, and imaging. Radiographs may initially result as normal; X-ray should be performed in two orthogonal planes with an anterior-posterior view, bilaterally for comparison with the opposite hip on the same image. MRI is more sensitive but is best performed as a second option in doubtful cases. Treatment is generally conservative, but sometimes surgery is mandatory for resolution.

### 2.2. Rheumatic Conditions

Reactive arthritis is an immune-mediated synovial inflammation that follows a few weeks after a bacterial or viral infection. The most common pathogens involved are enteric agents such as *Yersinia enterocolitica*, *Campylobacter jejuni*, *Shigella flexneri*, *Salmonella* spp., and respiratory viruses as *Rubella viru*s [3]. There is no specific diagnostic test, so diagnosis is only based on clinical characteristics and patient history. Reactive arthritis usually is self-limiting, resolving within 6 months, but development of a chronic form can occur [37]. Reactive arthritis of the hip joint in young children (mean age 3–8 years) is also known as transient synovitis. It is a self-limiting disorder, even if recurrences are possible, usually subsequent to additional viral infections. Treatment is symptomatic with NSAIDs and rest for a few days [38].

Rheumatic diseases are usually characterized by subacute onset, especially JIA, that is the most common rheumatic disease in children. However, among different JIA subtypes, systemic juvenile idiopathic arthritis (sJIA) may have an abrupt onset. Patients with sJIA present a septic appearance with arthritis in one or more joints with or preceded by quotidian fever (fever that rises to ≥39 °C once a day and returns to ≤37 °C between fever peaks and last at least 2 weeks) and accompanied by one or more of the following: evanescent erythematous rash that characteristically coincides with fever peaks, generalized lymph node enlargement, hepatomegaly and/or splenomegaly, and serositis. Laboratory investigation in sJIA shows leukocytosis (with neutrophilia), high ESR and CRP levels, thrombocytosis, and microcytic anemia [39]. Usually, the diagnosis is done after several days or weeks with typical findings and failure to respond to antibiotics.

Acute arthritis and polyarthralgia, or more specifically migratory polyarthritis, usually with overlying warm and red skin and with severe pain, are highly suggestive for acute rheumatic fever, a sequel of a group A streptococcal pharyngeal infection that typically occurs weeks after the precipitating infection. Diagnosis is confirmed by signs included in the Jones diagnostic criteria (carditis, chorea, erythema marginatum, subcutaneous nodules, fever, high ESR and CRP, and PR prolongation at EKG) and the laboratory evidence of a recent group A streptococcal infection proven by positive throat swab or increase of ASO titer [40]. In this case, arthritis is self-limiting in 2–3 weeks and has an excellent response to salicylates, but the diagnosis is important to exclude and prevent cardiac sequelae.

Acute arthritis is also one of the main features of Henoch-Schönlein purpura, the most common pediatric vasculitis. The patient may have an asymmetric oligoarthritis or a symmetric polyarthritis, mostly of large joints, always associated with palpable purpura (mandatory condition), mainly affecting the lower limbs and buttocks, and/or abdominal pain with possible severe evolution (such as perforations and invaginations) and/or renal involvement (1/3 of cases). Systemic symptoms such as fever and asthenia may precede or be concomitant to the classic ones. Arthritis may precede the typical purpura by hours or days, and only the appearance of the latter allows the diagnosis to be made [41]. Among vasculitides, arthritis is also one of the associated features of Kawasaki syndrome (KS), reported in 30% of cases; it develops in the first week of illness, tends to involve multiple joints, and is usually associated with a severe multisystemic disease and the possible development of coronary artery aneurysm. The arthritis heals without any sequelae [42].

Arthritis is the most common inflammatory bowel disease (IBD) extra-intestinal manifestation, arising in about 40% of IBD patients. These arthropathies, also named enteropathic arthritides, manifest mainly as either axial (e.g., sacroiliitis and ankylosing spondylitis) or peripheral spondyloarthritis (SpA). The axial involvement, characterized by low back pain and morning and rest stiffness, is more common in patients with Crohn’s disease (5–22%) than in those with ulcerative colitis (2–6%), and is strongly associated with uveitis and HLA-B27 positivity. Its onset may precede the enteritis, but there is no relation between the course of the two inflammatory conditions. Peripheral SpA are further categorized into two types. Pauciarticular (Type 1) peripheral arthritis presents as acute, asymmetrical arthritis involving less than five joints (mainly the large ones). It is self-limited, with episodes lasting less than 10 weeks, and correlates with intestinal-IBD activity. Hence, IBD treatment promotes also the improvement of arthritis. Polyarticular (Type 2) peripheral arthritis presents as symmetric arthritis involving small joints. It is unrelated to IBD activity and hence may precede the IBD diagnosis by years. Therefore, all patients with SpA need to be screened for associated subclinical gut inflammation through fecal calprotectin [43,44,45].

Joint pain is one of the most common complaints in children with lupus (jSLE). Malar rash, antiribonucleoprotein (anti-RNP) antibodies, anemia, and thrombocytopenia are all findings associated with arthritis in jSLE. Acute arthritis is usually a mild, self-limited and non-erosive peripheral arthritis, present at time of disease onset, mimicking JIA. Chronic rheumatoid factor–positive erosive arthritis (also named rhupus) and Jaccoud’s arthropathy are rare in jSLE [46]. Diagnosis requires that the SLICC criteria or the more recent EULAR/ACR SLE classification criteria are met [47]. Arthralgia and arthritis, more often with effusion of small joints, are also common in Sjogren syndrome and juvenile Mixed Connective Tissue Disease; in these cases, the presence of other peculiar symptoms (e.g., Raynaud phenomenon or sicca syndrome) and specific autoantibodies guide the diagnosis [48].

Different from “classic” sarcoidosis (usually without hilar lymphadenopathy and interstitial fibrosis) and typical of preschool age are the granulomatous auto-inflammatory diseases including the familial form known as Blau syndrome (BS, OMIM 186580) and the sporadic form known as early-onset sarcoidosis (EOS, OMIM 609464); both are caused by mutations in the NOD2/CARD15 gene and characterized by the clinical triad of granulomatous recurrent panuveitis, dermatitis, and symmetric arthritis with large, boggy synovial effusions and tenosynovial cysts particularly affecting the wrists and ankles. The diagnosis of EOS/BS can be supported by the demonstration of non-caseating granulomas within skin, synovial, or conjunctival biopsies, and the presence of NOD2 mutations [49].

Finally, arthritis may occur in patients affected by chronic nonbacterial osteomyelitis (CNO), a rare inflammatory bone disorder that usually occurs in children and young adults. In 30% of cases, CNO involves the adjacent joint with the presence of exudate, synovial thickening, and/or damage to the articular cartilage. The diagnosis is of exclusion and often bone biopsy is mandatory to rule out malignancies. In selected cases, clinical features, the presence of multifocal lesions, and radiologic features may help in diagnosis, avoiding bone biopsy [50].

### 2.3. Tumors

Tumors may present with bone pain and articular involvement. Malignant bone tumors usually have a localised focus; pain may wax and wane over time, and usually first appears after a minor trauma, but it can also be explosive. Skin can be tender, hot, and reddish, and sometimes there is also fever; laboratory tests can show elevated inflammatory markers (e.g., in Ewing sarcoma), but usually are normal. Differential diagnosis needs radiologic evaluation. Plain radiograph is a helpful tool that can be part of the first line work-up and has a characteristic appearance. Osteosarcoma, for example, usually shows radiologic destruction of the normal trabecular bone pattern, indistinct margins, and no endosteal bone response. Ewing sarcoma has a “permeative” or “moth-eaten” pattern on imaging with very poorly defined margins [51]. Final diagnosis, however, always has to rely on a bone biopsy. Leukemia may have a mild onset with musculoskeletal symptoms such as functional impairment and joint pain, also at night. Laboratory tests can be initially normal but become abnormal after a period with cytopenia of at list two lines [52]. Benign tumors can also be considered in the differential diagnosis of septic arthritis. Osteoid osteoma is a benign bone-forming tumor, usually involving the extremities. Typical clinical manifestations include bone pain not necessarily related to activity, which is usually relieved by nonsteroidal anti-inflammatory drugs [53]. The pathognomonic imaging sign is the small radiolucent nidus with sclerotic margins [54]. Pigmented villonodular synovitis, also known as tenosynovial giant cell tumor, is a benign tumor characterized by hypervascular proliferation of synovium that may also destroy the adjacent bone. Usually there is only one joint affected, especially the knee or the ankle, while shoulder, hip, elbow, and wrist involvement are less common [55] The classical clinical presentation is characterized by episodes of painful arthritis and spontaneous hemarthrosis. Imaging is very helpful: ultrasonography is able to identify hypervascular areas, but MRI is the gold standard for diagnosis with a focus on hemosiderin deposition [56].

## 3. Conclusions

Acute arthritis is a common condition in childhood that potentially leads to severe complications and life-threatening consequences. Doubtful cases, especially in the presence of fever, should be managed as a bacterial infection until proven otherwise and promptly referred to secondary pediatric care. Current tools employed to guide the clinician rely on non-specific inflammatory markers, radiologic imaging, and arthrocentesis. A rapid onset and fast worsening of findings such as fever (>38°), severe pain, swelling, and limited range of motion of an inflamed joint are suggestive for septic arthritis. Synovial fluid and blood samples should be promptly collected for culture and PCR analyses, possibly without a delay in starting an empiric antibiotic treatment. Laboratory tests are non-specific: Acute-phase reactants are usually elevated and associated with leukocytosis in septic arthritis but also in inflammatory arthritis, while at the same time they are normal or only mildly elevated in the case of *Kingella kingae* infection. Radiography is helpful in identifying concomitant osteomyelitis and evaluating other conditions in the differential diagnosis (e.g., trauma, neoplasia). MRI should be considered in any doubtful case, when arthritis does not improve after 48 h of antibiotics, in the case of suspected concomitant osteomyelitis. Insidious onset, nocturnal bone pain, pallor, unexplained bruising, liver or spleen enlargement, low fever, and abnormalities in complete blood count need an accurate investigation since they are suggestive for neoplasms. Remittent or intermittent fever, history of rash, weight loss, abdominal pain and ocular abnormalities, lymphadenopathy, and serositis or long-lasting arthritis are indicative of an inflammatory condition; therefore, referral to a pediatric rheumatologist is most appropriate, with outpatient review within 1–2 weeks. If history or clinical and imaging are suggestive for trauma or osteochondrosis, the pediatrician can require orthopedic evaluation. Differential diagnosis can be difficult in some cases and has to be guided always by clinical evaluation in the first place; laboratory tests and imaging are useful in the second place to confirm a diagnostic suspicion. If there is any doubt of a bacterial infection, broad spectrum antibiotic treatment should always be performed as soon as possible, to avoid any sequelae.
